# Genetic and pathogenic characterization of SARS-CoV-2: a review

**DOI:** 10.2217/fvl-2020-0129

**Published:** 2020-08-26

**Authors:** Afsane Bahrami, Gordon A Ferns

**Affiliations:** 1 ^1^Cellular and Molecular Research Center, Birjand University of Medical Sciences, Birjand, Iran; 2 ^2^Brighton & Sussex Medical School, Division of Medical Education, Falmer, Brighton, Sussex BN1 9PH, UK

**Keywords:** betacoronavirus, remidisvir, SARS-CoV-2, spike surface glycoprotein

## Abstract

The first case of Coronavirus disease 2019 (COVID-19) caused by severe acute respiratory syndrome coronavirus 2 (SARS-CoV-2) was reported in December 2019. This virus belongs to the beta-coronavirus group that contains a single stranded RNA with a nucleoprotein within a capsid. SARS-CoV-2 shares 80% nucleotide identity to SARS-CoV. The virus is disseminated by its binding to the ACE2 receptors on bronchial epithelial cells. The diagnosis of COVID-19 is based on a laboratory-based reverse transcription polymerase chain reaction (RT-PCR) test together with chest computed tomography imaging. To date, no antiviral therapy has been approved, and many aspects of the COVID-19 are unknown. In this review, we will focus on the recent information on genetics and pathogenesis of COVID-19 as well as its clinical presentation and potential treatments.

Coronaviruses (CoVs) are enveloped, positive-sense RNA viruses that are primarily responsible for infections of the upper respiratory and digestive tract [1,2]. CoV originates from the Latin word corona. The peplomers on the surface of the virus create an image reminiscent of a solar corona on electron microscopy. These viruses associated with severe acute respiratory syndrome CoV (SARS-CoV) and Middle East respiratory syndrome CoV (MERS-CoV) [1,2], that occurred as outbreaks in 2002 and 2013, respectively, and both have been associated with severe complications, that include, severe pneumonia and bronchiolitis [1]. CoVs belong to the subfamily *Coronavirinae*, family *Coronaviridae* and order *Nidovirales*, which can be divided into four genera: alphacoronavirus (α-CoVs), betacoronavirus (β-CoVs), gammacoronavirus (γ-CoVs) and deltacoronavirus (δ-CoVs) [3].

There are seven types of CoVs known to cause infections in humans including 229E (α-CoVs), NL63 (α-CoVs), OC43 (β-CoVs), HKU1 (β-CoVs), MERS-CoV (β-CoVs), SARS-CoV (β-CoVs) and recent novel β-CoVs (SARS-CoV-2; Figure 1) [4–6]. The three last viruses can affect respiratory, gastrointestinal, hepatic and nervous systems, and may cause life-threatening respiratory and multiple organ failure and death in some severely affected patients [7,8]. α-CoVs and β-CoVs have been responsible for a heavy disease burden on livestock; for example, an HKU-2 bat origin, swine acute diarrhea syndrome-coronavirus emerged in the swine population in 2017 in Guangdong in China [9].

**Figure 1.**
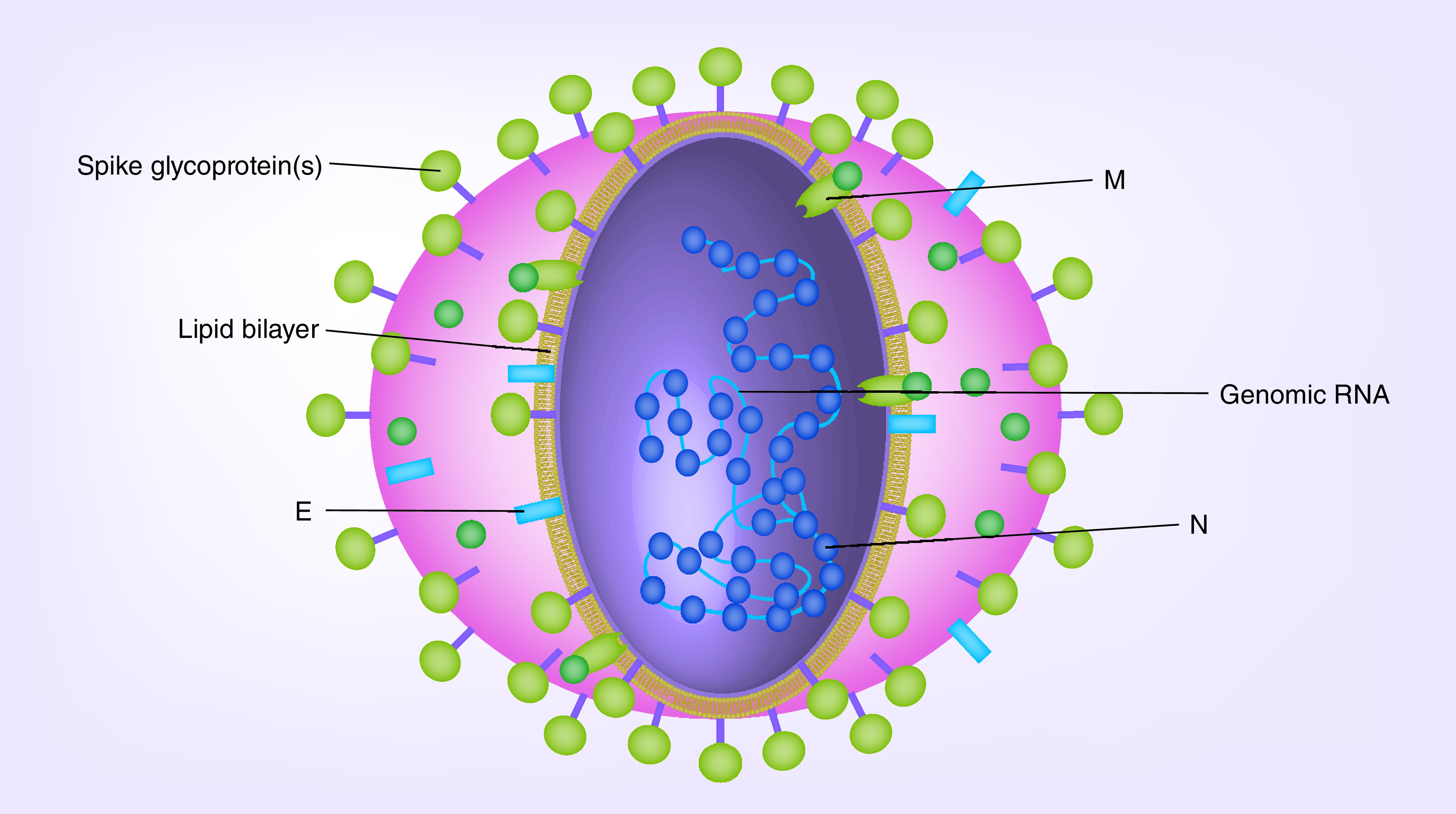
Coronavirus particle. E: Envelope protein; M: Membrane protein; N: Nucleocaspid.

The first case of a coronavirus disease 2019 (COVID-19) infection was reported in December 2019 in Wuhan City, Hubei Province, China and after that it has spread globally to being declared to be a pandemic by the WHO in 2020 [10]. SARS-CoV-2, was first isolated from patients with pneumonia of unknown etiology, and may have originated from a ‘wet market’ where wild animals were being sold in Wuhan. But, Wuhan seafood market probably only served as an amplifying hotspot and not is source of novel virus spreading globally. High levels of SARS-CoV-2 transmission have now been reported in the USA, Italy, Iran, Germany and France. More than 12 million affected cases and 500,000 deaths globally have been reported until 10 July. The estimated mean reproduction number (*R0*) for COVID-19 without intervention, is around 3.28 which is higher than for SARS-CoV [11].

## The SARS-CoV-2 genome & structure

**Figure 2.**
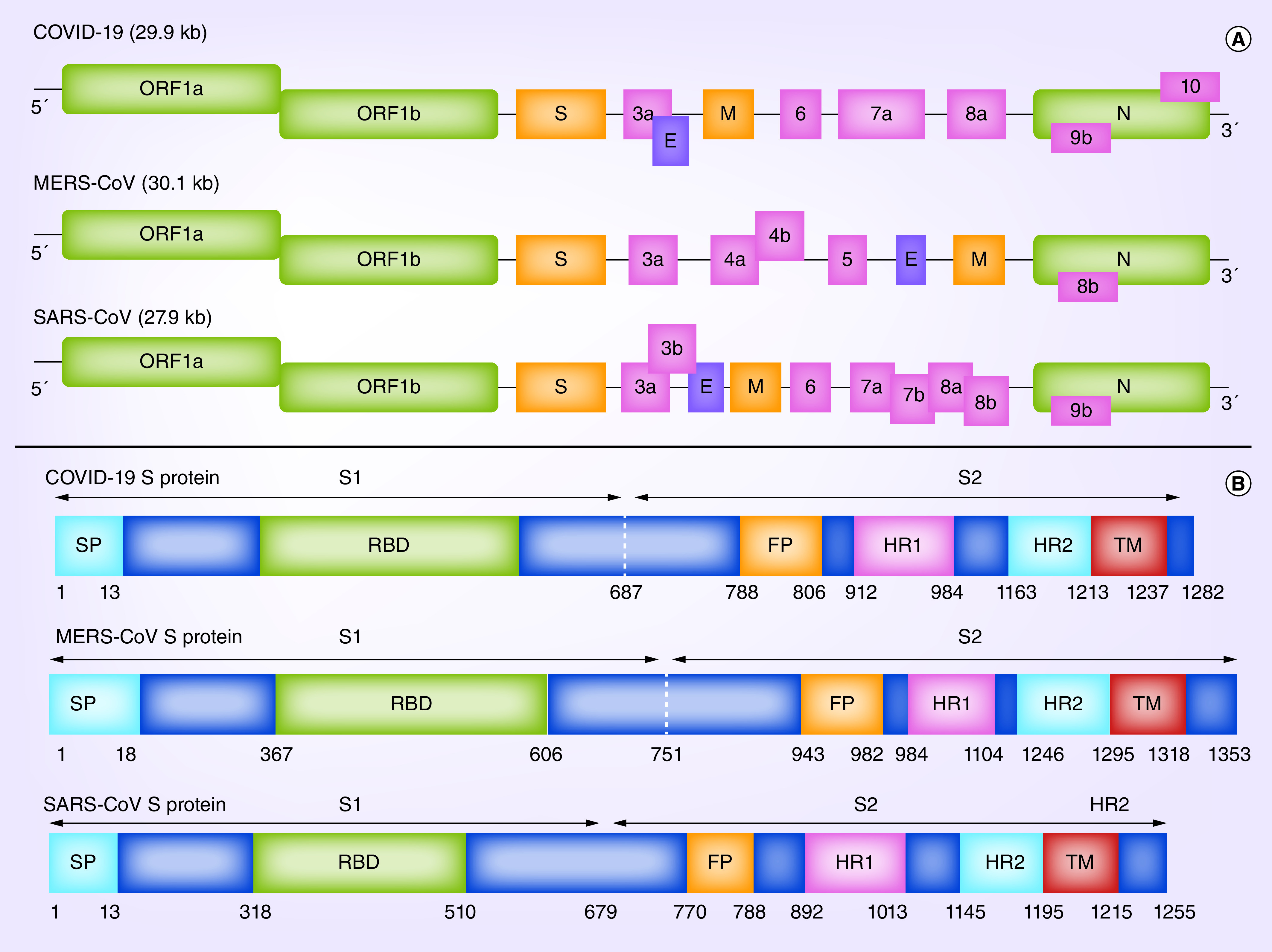
Genome organization of coronaviruses and spike protein. **(A)** The coronavirus structure including the 5′-UTR, orf 1a/b, structural proteins including S, E, M and N glycoproteins as well as several accessory proteins (such as orf 3, 6, 7a, 7b, 8 and 9b) and the 3′-UTR. **(B)** Comparison of the S proteins of SARS-CoV, MERS-CoV and SARS-CoV-2. SP, receptor-binding domain, FP, HR1/2 and TD. 3′-UTR: 3′-untranslated region; 5′-UTR: 5′-untranslated region; COVID-19: Coronavirus disease 2019; E: Envelope; FP: Fusion peptide; HR1/2: Heptad repeat 1/2 MERS-CoV: Middle East respiratory syndrome-coronavirus; ORF: Open reading frame; S: Spike; SARS-CoV: Severe acute respiratory syndrome-coronavirus; SP: Signal peptide; TD: Transmembrane domain

All the CoVs are positive-stranded RNA viruses with a polycistronic genome nearly 29.9 kb in length with 6–11 open reading frames (ORFs), encoding several nonstructural proteins (ORF1a and ORF1b, that are processed into 15 nsp proteins) at the 5′-end plus four structural proteins (spike surface glycoprotein [S], envelope [E], matrix [M] and nucleocapsid [N]) and multiple lineage-specific accessory proteins (i.e., ORF3a, ORF6, ORF7a, ORF7b, ORF8 and ORF10 in SARS-CoV-2) at the 3′-end (Figure 2A) [3,12,13]. The SARS-CoV-2 virus also encodes other poly-proteins, nucleoproteins and membranous proteins, in other words RNA polymerase, helicase and various proteases [13,14].

**Figure 3.**
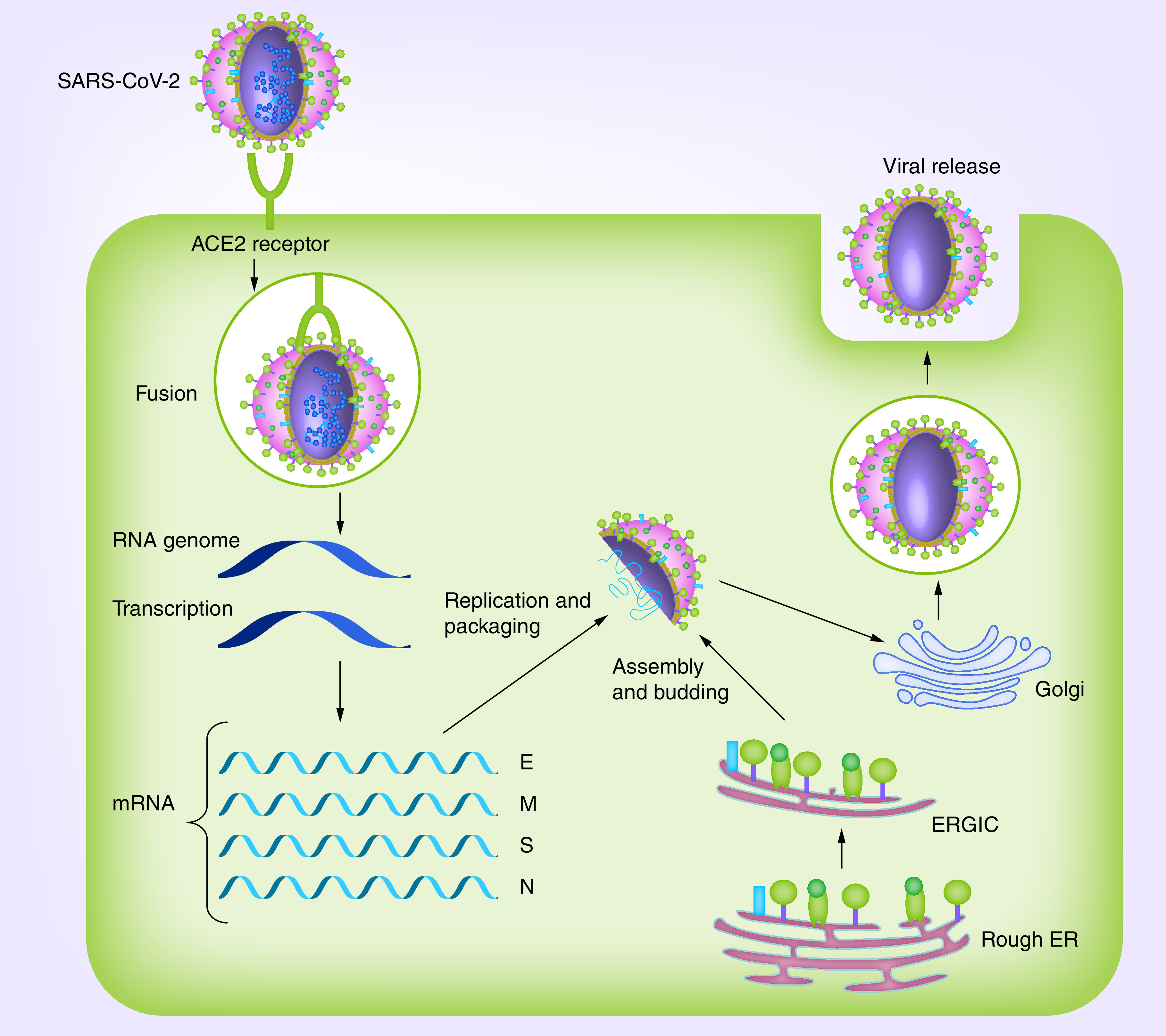
SARS-CoV-2 life cycle. Spike glycoprotein bind to ACE2 receptor to assist virus entrance. Subsequent to the fusion of viral and plasma membranes, virus RNA undergoes replication and transcription. After the production of SARS-CoV-2 structural proteins, nucleocapsids are assembled in the cytoplasm and, followed by budding into the lumen of the ERGIC. New virions are released via vesicles. E: Envelope protein; ER: Endoplasmic reticulum; ERGIC: Endoplasmic reticulum–Golgi intermediate compartment; M: Membrane protein; N: Nucleocaspid; S: Spike.

The initial infection requires viral entry into the host cell. The S-glycoprotein mediates binding of the virus to the human cell surface receptors, followed by fusion of the virus and host cell membranes to assist viral entrance (Figure 3) [15,16]. The S-glycoprotein is a structural protein that accounts for the crown-like shape of the CoVs. The 1200 AA length S-protein (approx. 180 kDa) is a member of class-I viral fusion proteins [17,18]. It is expressed on the surface of the virus as a trimetric protein and also determines the host tropism and pathogenesis [19–21]. The S-glycoprotein can be cleaved by host proteases to an N-terminal S1-ectodomain and C-terminal S2-membrane-anchored protein (Figure 2B) [22]. In several CoVs, the higher amplification of S-protein at the cell membrane can also facilitate membrane fusion of infected cells with adjacent uninfected cells, and this leads to the formation of giant or multinucleated cells (syncytia) and further spread of the virus between cells [23–25]. The CoVs S1-protein includes a receptor-binding domain (RBD), which binds to the host cell via receptors that include the ACE2 receptor [26]. SARS-CoV uses the ACE2 receptor for entry [26] and uses the serine protease, cathepsins and TMPRSS2, for S-protein priming [24,27]. Of 14 AA residues in the S1 subunit of SARS-CoV, eight residues are highly conserved in SARS-CoV-2, suggesting that the ACE2 receptor is also used for cell entry by this virus [28]. Labeling studies have confirmed this [4,29]. The S-glycoprotein of SARS-CoV-2 has a 3D structure within the RBD domain that conserve the van der Waals interactions [4]. The 394 glutamine residue of the SARS-CoV-2's RBD domain is ligated via the important lysine 31 residue of the human receptor, ACE2 [28].

An E-protein is involved in the assembly and release of virus-like particles [30–32]. M-protein provides the shape of the viral envelope and binding to nucleocapsid [33], whereas, the N-protein binds to the CoV RNA genome, building the nucleocapsid to support replication–transcription complex [34]. Interaction between M and N-proteins stabilize the nucleocapsid and the internal core of virus, and, eventually, enhance the assembly of the virion particles [23,35,36]. M-, E- and S-proteins then enter into the endoplasmic reticulum–Golgi intermediate compartment complex and constitute the viral envelope [36]. Accessory proteins appear to promote the adaptation of CoVs to human host cells [37].

Genomic analysis of ten genome of SARS-CoV-2 isolated from nine patients demonstrated 99.98% nucleotide identity [38]. Another report found that 99.8–99.9% sequence similarity in sample of five infected patients [39]. Phylogenetic analysis demonstrates that SARS-CoV-2 shares 50 and 80.0% nucleotide identity to MERS-CoV and SARS-CoV, respectively [8,13,38].

The SARS-CoV-2 constitutes a clade among the sub-genus sarbecovirus [40]. Bioinformatics analysis of the viral genome from one COVID-19 patient shared 89 and 82% sequence similarity with bat SARS-like-CoVZXC21 and human SARS-CoV, respectively [41]. However, the external subunit of Spike RBD of SARS-CoV-2 has only 40% amino acid (AA) identity with other SARS-associated CoVs [41]. The S-protein of SARS-CoV-2 is longer (1282 AA) than for other viruses such as SARS-CoV (1255 AA) and Bat SARS-like CoVs (1246 AA). The S-glycoprotein of SARS-CoV-2 has been found to have three short insertions at the N-terminal end, with four variations in the receptor binding site within the RBD compared with SARS-CoV [42]. Notably, SARS-CoV-2 ORF3b codifies a new short protein. Moreover, its novel ORF8 sequence possibly encode a secreted protein with an α-helical structure with a β-sheet(s) consisting of six strands [41]. The high levels of genetic identity (96.3%) between the SARS-CoV-2 and Bat-CoV RaTG13 does not indicate the precise variant that may have led to the outbreak in humans, although it has been suggested that the likelihood that the novel CoV has derived from bats is very probable [43]. SARS-CoV-2 and RaTG13 differ with respect to the number of major genomic properties, in which SARS-CoV-2 harbors a polybasic (furin) cleavage site insertion at the connection of the two subunits of the S-protein, S1 and S2 [44].

The N-protein is hidden within phospholipid bilayers and coated via two distinctive forms of S-proteins including the spike glycoprotein trimmer which is present in all CoVs, as well as the hemagglutinin-esterase (*HE*) solely found in certain CoVs. For instance, SARS-CoV-2 does not appear to have the *HE* gene. The M and E proteins are found inside the S-glycoproteins within the viral envelope [45]. The S, E, M, N and ORF3a genes of SARS-CoV-2 are predicted to be 3822, 228, 669, 1260 and 828 nucleotides in length, respectively. Moreover, SARS-CoV-2 has been predicted to contain an ORF8 gene, of 366 nucleotide size, situated between the M and N corresponding ORF genes [45].

The SARS-CoV sequence reveals serine substituting for glycine in the residue at position 543 of the nsp3 protein in Bat SARS-like and SARS-CoV. This AA substitution could promote local stiffness of the polypeptide chain for steric impact and potency of the serine side-chain to constitute H-bonds. Beside, serine is a nucleophile that can establish structural environments, like those at active sites of enzyme. Mutations in the nsp3 protein were reported to affect the replication of SARS-CoV-2 in infected cells [46,47]. It has been reported that the single N501T variant in SARS-CoV-2's S-protein may enhance binding affinity for the ACE2 cellular receptor [28]. Furthermore, a single N439R mutation in SARS-CoV-2 RBD promotes its ACE2-receptor binding and, thus potentially enhances human-to-human transmission [48,49]. By studying the crystal structure of SARS-CoV-2 RBD binding to the human ACE2 receptor has shown that the ACE2 receptor-binding ridge in SARS-CoV-2 RBD results in a more compact conformation, leading structural alterations at the RBD/ACE2 interface versus the SARS-CoV [48]. Overall, SARS-CoV-2 binding affinity for ACE2 is 10–20-times greater than for other SARS-associated CoVs [50].

A missense mutation at the 614 position of S protein (aspartate to glycine, D614G mutation), in the spike protein of SARS-CoV-2, which has emerged as a predominant clade in Europe (66% sequences) and is spreading worldwide (44% sequences). The D614G mutation promotes viral infectivity and transduction of multiple human cell types and mitigates neutralization sensitivity to individual convalescent sera [51–54].

## SARS-CoV-2 pathogenesis

Lipids play important roles at different stages in the CoVs life cycle. CoVs recruit intracellular membranes of the host cells to produce new compartments, or double membrane vesicles, which are used for the replication of the virion particle genome [55]. Recently, an important lipid processing enzyme, known as cPLA2 α has been reported to be related to the formation of double membrane vesicle and CoV’s amplification [56]. It has been demonstrated that the enzyme, phospholipase A2 group IID, is involved in anti-inflammation or proresolving lipid mediator regulation which may lead to worse outcomes in a SARS-CoV infection animal model by modulating the immune response [57].

It has been shown that there is a distinct insert that includes basic AAs in the S1/S2 priming loop of SARS-CoV-2, which is not found in SARS-CoV or any SARS-associated CoVs. It may substantially alter the entry pathway of SARS-CoV-2 compared with other viruses of the β-CoVs lineage B [58]. In a recent report it was shown that SARS-CoV-2's S-protein entry into 293/human ACE2 receptor cells is primarily mediated via endocytosis, and that PIKfyve, a TPC2 and cathepsin L are crucial for virus entry. PIKfyve is the key enzyme in the early endosome involved in the synthesis of PI(3,5)P2 and its main downstream effector, TPC2. The S protein of SARS-CoV-2 could also stimulate syncytia in 293/human ACE2 cells independently of exogenous protease [59].

In a study of 452 SARS-CoV-2 infected patients, it was found that severely affected cases had lower numbers of blood lymphocytes, percentages of monocytes, basophils and eosinophils as well as increased leukocytes numbers and neutrophil-lymphocyte-ratio. In most patients with unfavorable progression of COVID-19, elevated concentrations of infection-associated markers and inflammatory cytokines was observed. The frequency of T cells was significantly lower, and less effective in severely affected subjects. Both T helper (Th) cells and suppressor T cell numbers in patients with COVID-19 were below the reference range. The percentage of naive helper T cells was increased, and memory helper T cells and regulatory T cells reduced in severe conditions [60]. Furthermore, simultaneous to the infection with SARS-CoV-2, CD4^+^ T lymphocytes are quickly over-activated to switch to the pathogenic Th1 cells producing GM-CSF. The cytokines environment activates inflammatory CD14^+^CD16^+^ monocytes, leading to over-expression of IL-6 and enhances the inflammatory response. Regarding the increased infiltrations of inflammatory cells that have been found in lungs of severe SARS-CoV-2 infected patients [61,62], these population of abnormal and noneffective pathogenic Th1 cells and inflammatory granulocytes may go to the pulmonary circulation and by immune stimulation, lead to functional impairment of the lungs and eventually death [63].

Inflammasomes are very large intracellular poly-protein signaling complexes which are constitute in the cytosol as an inflammatory immune reaction to endogenous danger stimuli [64]. NLRP3 responds to wide spectra of pathogens and endogenous signals, and is involved in the molecular pathway of various auto-inflammatory disorders [65]. It has been reported that the SARS-CoV can induce the NLRP3 inflammasome in macrophages through ORF8b. Whereas SARS-CoV infects macrophages or monocytes, sufficient ORF8b may be present to impact on the autophagy-lysosome pathway, and NLRP3 inflammasomes. SARS-CoV replicates efficiently in lung epithelial cells. These cells also amplify NLRP3 and support assembly of NLRP3 inflammasomes. In SARS-CoV patients, the full effect of the ORF-8b on these inflammatory cascades was observed in the lung epithelium. Interestingly, ORF8b may be involved in the ‘cytokine storm’ or ‘cytokine cascade’ and inflammasome induction which happens within intensive SARS-CoV infection [66].

SARS-CoV-2 infection stimulates the immune response in two stages. In the early stages, a particular adaptive immune response is necessary to eradicate the virus and to impede progress to a more severe condition. The protective immune response at this phase requires that the host should have excellent general health and a suitable genetic context which provides antiviral immunity [67]. Although, when the immune response protection is disabling, virus will disseminate and great damage to the affected tissues occurs, particularly in organs with a high levels of ACE2 receptor expression. The injured cells activate innate inflammation within the lungs which is mainly mediated through pro-inflammatory macrophages/monocytes. Lung inflammation is the major reason for the fatal respiratory disease at the severe stage of COVID-19 [68].

In viral infections, host antiviral microRNAs participate in the regulation of immune response to virus and are capable of targeting viral genes and interfere with replication, mRNA expression and protein translation of virion particle gene. Sardar *et al.* predicted the antiviral host-microRNAs specifically for COVID-19. They reported a list of six microRNAs related to COVID-19 including hsa-let-7a, hsa-miR101, hsa-miR126, hsa-miR23b, hsa-miR378 and hsa-miR98 which has been previously reported to be related to other viral infections, such as HIV [69].

## Clinical presentation

Virion particles spread from the respiratory mucosa, by binding to the ACE2 receptors on ciliated bronchial epithelial cells, and after that may engage with other cells [70]. In one report from Wuhan, the average incubation period of 425 SARS-CoV-2 infected patients was 5.2 days, but it this differed between individuals [71,72]. Until now, most patients with COVID-19 have initially presented with mild manifestations in other words dry cough, sore throat and fever which spontaneously resolve. Although, some patients have developed other more severe disease such as organ failure, septic shock, pulmonary edema, dyspnea, myalgia, fatigue and acute respiratory distress syndrome [73]. In contrast to SARS-CoV, patients infected with SARS-CoV-2, development of upper respiratory tract signs and manifestations are less common, suggesting that SARS-CoV-2 may target cells in the lower airway [74]. Among cases with severe dyspnea, more than 50% have required intensive care. Some COVID-19 cases do not present with fever or radiologic abnormalities on admission, which makes initial diagnosis difficult [75].

The main characteristics of COVID-19 on preliminary CT examination including bilateral multi-lobar ground-glass opacities with a peripheral/posterior distribution and patchy consolidation, primarily in the lower lobes and fewer inside the right middle lobe [76]. The main reported laboratory test abnormalities in cases with severe COVID-19 infection include: increased levels of liver enzymes (LDH, ALT and AST), total bilirubin, creatinine, cardiac troponin, D-dimer, prothrombin time, procalcitonin and CRP [77].

The histology of liver specimens of SARS-CoV infected patients have revealed a remarkable liver injury with an increase in mitotic cells, along with eosinophilic bodies as well as balloon-like hepatocytes [78]. Cardiac involvement is another prominent manifestation of COVID-19 and is closely related to a poor outcome [79]. In a recent systematic review, the incidence rate of diarrhea varied from 2 to 50% in COVID-19 patients. It may develop earlier, or following the respiratory symptoms. Findings of several studies showed that viral RNA shedding is detect for a longer time period compared with nasopharyngeal swabs [50].

In an investigation on 1099 COVID-19 patients, of whom 23.7% had severe disease with comorbidities of hypertension, 16.2% diabetes mellitus, 5.8% coronary heart diseases and 2.3% cerebrovascular disease [80]. Another study, of 140 patients with COVID-19, found that 30% and 12% had history of hypertension and diabetes, respectively [81]. Analysis of 487 COVID-19 cases, showed that older age (odds ratio [OR] = 1.06; 95% CI: 1.03–1.1), male gender (OR 3.7; 95% CI: 1.7–7.7) and hypertension as a comorbidity (OR 2.7; 95% CI: 1.3–5.6) are related with more severe disease on admission [82]. Moreover, patients with cancer were more vulnerable to severe events from COVID-19 such as admission to the intensive care unit needing invasive ventilation, or death [83].

It has been reported that the highest viral load in throat swabs occurs at the time of development of symptoms. However, viral shedding was reported to occur before the onset of symptoms, and a major proportion of transmissibility happened before first symptoms in the index case [84]. Furthermore, severe COVID-19 cases tend to have an increased viral load and a long virus-shedding time [85].

## Diagnosis

At present, the diagnosis of COVID-19 is largely based on guideline agreement that includes laboratory tests and chest CT imaging technique [75,86]. PCR testing of asymptomatic or mild symptomatic contacts can be used in the evaluation of peoples who have been in contact with a COVID-19 case [87], and the WHO has not accepted the results of a chest CT without RT-PCR conformation in the diagnosis of COVID-19 [88].

Chest CT is a routine imaging tool for the diagnosis of pneumonia, which is relatively easy and rapid to perform. Chest CT shows typical radiographic characteristics in almost all COVID-19 cases, such as peripheral/posterior distribution and patchy consolidation, and/or interstitial alterations with a peripheral distribution, so may provide benefit for diagnosis of COVID-19 [89].

Respiratory tract samples were collected for the diagnosis and screening of patients with SARS-CoV-2 pneumonia; in the 5–6 days of the initiation of symptoms, patients with COVID-19 have increased viral loads in their upper and lower respiratory tracts [90,91]. For suspected cases, real-time fluorescence (RT-PCR) was performed to detect the positive nucleic acid of SARS-CoV-2 in sputum, throat swabs and secretions of the lower respiratory tract specimens [92]. A nasopharyngeal and/or an oropharyngeal swab are frequently recommended for screening or diagnosis of early infection [10,93]. A single nasopharyngeal swab has become the preferable swab as it is well-tolerated by the patient and safer for the operator.

Serological testing detects presence of IgG, IgM or both. A positive elucidation has been defined as a positive lgM, or convalescent sera with a higher lgG titer >four-times in comparison with the acute phase. SARS-CoV-2 IgG and IgM are detected in whole blood, plasma, serum or specimens. Antibodies increase late in the course of illness; the mean duration of SARS-CoV-2 IgM antibody detection was reported to be 5 days, whereas IgG detection about 2 week following the appearance of symptoms [94]. In contrast to respiratory samples which may disturb from false-negative results because of the sampling factors, the presence of antibodies in blood uniformly is detectable. Specimens are easier to gather versus respiratory samples, such as fewer risks to the operator. The serological assay is very easy, rapid, availability of ELISA platforms, requires no instrumentation and can provide results in just 15 min [95].

## Treatment

Based on the recommendation of WHO, COVID-19 management protocols have mostly highlighted infection prevention, patient early detection and monitoring, and best supportive care [96,97]. No specific antiviral treatment is currently recommended for COVID-19 due to lack of evidence. Many treatment regimens have been assessed for COVID-19, some showing promising preliminary results. A total of 2531 trials on COVID-19 have been registered to date in the clinicaltrial.gov (updated 10 July 2020). Several pharmacotherapeutic agents have been used including lopinavir/ritonavir, hydroxychloroquine and IFN-β-1A (Table 1).

**Table 1.** Clinical trials for the treatment of coronavirus disease 2019 (updated at 10 July 2020).

Intervention	Phase	Status	References^†^
– Lopinavir/ritonavir	II	Recruiting	NCT04330690
– Lopinavir/ritonavir – Hydroxychloroquine sulfate	II	Terminated	NCT04307693
– Interferon-β 1a – Lopinavir/ritonavir – Single dose of hydroxychloroquine	IV	Enrolling by invitation	NCT04350671
– Umifenovir – Interferon-β 1a – Lopinavir/ritonavir – Single dose of hydroxychloroquine – Standards of care	IV	Enrolling by invitation	NCT04350684
– Hydroxychloroquine – Lopinavir/ritonavir – Interferon-β 1a – Interferon-β 1a	IV	Completed	NCT04343768
– Lopinavir/ritonavir – Hydroxychloroquine sulfate – Baricitinib (janus kinase inhibitor) – Sarilumab (anti-IL-6 receptor)	II	Recruiting	NCT04321993
– Hydroxychloroquine	III	Recruiting	NCT04345692
– Hydroxychloroquine + camostat mesylate – Hydroxychloroquine+ azithromycin	III	Recruiting	NCT04355052
– Hydroxychloroquine – Azithromycin	II	Recruiting	NCT04329832
– Clevudine – Hydroxychloroquine	II	Recruiting	NCT04347915
– Hydroxychloroquine sulfate – Azithromycin – Chloroquine sulfate	III	Suspended	NCT04341727
– Chloroquine	II	Not yet recruiting	NCT04333628
– Standard care	III		
– Hydroxychloroquine – Azithromycin	III	Recruiting	NCT04334382
– Hydroxychloroquine	I	Recruiting	NCT04351620
– Hydroxychloroquine	III	Suspended	NCT04329611
– Camostat mesilate – Placebo – Hydroxychloroquine	IV	Not yet recruiting	NCT04338906
– Favipiravir – Placebo	III	Active, not recruiting	NCT04336904
– Usual care – Anakinra – Siltuximab – Tocilizumab	III	Recruiting	NCT04330638
– Tocilizumab Injection	II	Recruiting	NCT04317092
– Baricitinib	II/III	Not yet recruiting	NCT04340232
– Ruxolitinib	II/III	Not yet recruiting	NCT04348071
– Tocilizumab – Methylprednisolone	III	Not yet recruiting	NCT04345445
– Sildenafil citrate tablets	III	Recruiting	NCT04304313
– Deferoxamine	I/II	Recruiting	NCT04333550
– Naproxen – Standard of care	III	Not yet recruiting	NCT04325633
– Traditional Chinese medicine prescription	III	Not yet recruiting	NCT04323332
– Conventional medicines + traditional Chinese medicines granules – Conventional medicines	Not applicable	Recruiting	NCT04251871
– Dietary supplement: ascorbic acid – Dietary supplement: zinc gluconate – Dietary supplement: ascorbic acid and zinc gluconate – Other: standard of care	Not applicable	Enrolling by invitation	NCT04342728
– Losartan	IV	Recruiting	NCT04340557
– Captopril or enalapril – chloroquine	III	Not yet recruiting	NCT04345406
– Hyperimmune plasma	Not applicable	Completed	NCT04321421
– Blood donation from convalescent donor	–	Recruiting	NCT04351659
– Convalescent plasma	II	Recruiting	NCT04343755
– Convalescent plasma	II	Withdrawn (due to opening expanded access protocol)	NCT04325672
– rhACE2	Not applicable	Withdrawn (without CDE approval)	NCT04287686
– Allogeneic human dental pulp stem cells – Placebo	I/II	Recruiting	NCT04336254
– Allogeneic adipose-derived mesenchymal stromal cells – Placebo	I/II	Withdrawn	NCT04341610
– Biological: MSCs	I	Recruiting	NCT04252118
– Biological: NK cells, IL15-NK cells, NKG2D CAR-NK cells, ACE2 CAR-NK cells, NKG2D-ACE2 CAR-NK cells	I/II	Recruiting	NCT04324996
– Biological: NK cells	I	Recruiting	NCT04280224
– Biological: allogeneic NK transfer	I/II	Not yet recruiting	NCT04344548

† From Clinicaltrial.gov.

MSC: Mesenchymal stem cell; NK: Natural kiler.

### Chloroquine

Results from several *in vitro* and clinical studies demonstrated that chloroquine phosphate, an old agent for the treatment of malaria, had significant efficacy and acceptable safety for treatment of COVID-19 [98,99]. Findings of an open-label nonrandomized clinical trial among 22 infected patients indicated that hydroxychloroquine treatment significantly reduced viral load in COVID-19 cases and its effectiveness is promoted by azithromycin [99]. In a systematic review including six published articles highlighting the potency of chloroquine in attenuation the replication of SARS-CoV-2-associated virus [100]. But several other studies demonstrated no evidence of a strong antiviral function, or clinical benefit of the hydroxychloroquine for the treatment of patients with severe COVID-19 [101,102].

### Lopinavir–ritonavir

The combination of lopinavir/ritonavir (LPV–R) is extensively used for treating HIV-infected patients. LPV–R has been suggested for treatment of COVID-19. A total of 199 COVID-19 patients were randomly assigned to receive LPV–R (n = 99) or standard-care (n = 100). Treatment with LPV–R was not different from standard care regarding the time to clinical improvement, mortality rate at 28 days, as well as detection of viral RNA at different time points [103].

### Arbidol

Arbidol as a wide-spectrum antiviral compound that can inhibit viral fusion of influenza. In one study, 50 patients with laboratory-confirmed SARS-CoV2 were randomly divided into two arms: 34 cases received LPV–R (400 mg/100 mg, two per day) and 16 patients were administrated arbidol (0.2 g a; three per day). No difference was observed concerning fever duration between the two arms. 2 weeks after the intervention, no viral load was found in cases received arbidol, while the viral load was detectable in 44.1% of LPV–R group patients. Moreover, no adverse side effects were reported in either arm [104].

### Nelfinavir

Nelfinavir (NFV) is a potent HIV-1 protease inhibitor that received US FDA approval in 1997 for treatment of HIV infection. The antiviral activity of NFV against SARS-CoV-2 was reported in Vero E6 cells [105]. By using an integrative computational drug-discovery method, NFV was introduced as a potential inhibitor of SARS-CoV-2 main protease [106].

### Main protease inhibitor

The main protease of CoVs (Mpro) is an important protein necessary for the proteolytic maturation of the virion particle [107]. Therefore, targeting Mpro is considered to havepotential as a treatment for COVID-19 through suppression of the polypeptide cleavage virus [108–110]. Concerning the results of molecular docking, natural polyphenols such as hesperidin, rutin, diosmin, apiin and diacetyl-curcumin have been reported to have acceptable efficacy to target SARS-CoV-2 Mpro than NFV [111].

### Cytokine antagonists

Cytokine-directed antagonists, in other words adalimumab (TNF-α) and CMAB806 (IL-6) against SARS-CoV-2 have been evaluated in clinical trials. The variety of cytokines such as type-I IFN-I contribute to the ‘cytokine storm’ and pathology of SARS-CoV-2. Therefore, targeting the upstream origin of cytokine generation could be a promising therapeutic approach [112].

### Nucleoside analog

Utilizing an *in silico* model, it has been shown that antipolymerase agents including sofosbuvir, IDX-184, ribavirin (RBV) and remidisvir (GS-5734; RDV) can target RNA-dependent RNA polymerase of SARS-CoV-2 [113]. The first severe-infected patient with SARS-CoV-2 in the USA was cured by reception of intravenous RDV [114]. Due to adverse side effects, the appropriate dose of RBV in clinical setting should be given with caution.

### Convalescent plasma

In previous experience, for example in pandemic influenza A (H1N1), and avian influenza A (H5N1), passive immunization has been successful for treating of infectious complications [115]. A remarkable reduction in viral load and mortality was observed by using convalescent plasma therapy against severe acute viral respiratory infections, such as those created by CoVs [116]. Patients who have recovered from SARS-CoVs infection often have high titers of neutralizing antibody and may be a precious source of convalescent plasma. The FDA has also approved the administration of plasma from recovered individuals for treatment of severe COVID-19 patients [117].

### Monoclonal antibodies

SARS-CoV-specific human monoclonal antibody (mAb) can bind potently with SARS-CoV-2 region. But, some of the most powerful SARS-CoV-particular neutralizing antibodies (i.e., m396) that target the ACE2 binding site of SARS-CoV did not bind to SARS-CoV-2 S-protein, indicating that the disparity in the RBD of SARS-CoV and SARS-CoV-2 has an important effect impact on the cross-reactivity of these mAbs, and so novel mAbs that specifically target SARS-CoV-2 RBD need to be designed [112].

### Vaccine

Effective SARS-CoV-2 vaccines are urgently needed in order to reduce infection severity, viral shedding as well as human–human transmission, so assisting the control of the CoV outbreaks. Because S-protein and associated fragments, in other words RBD of SARS- and MERS-CoVs are the main targets for designing vaccines, it is speculated that homologous regions of SARS-CoV-2 can also be applied as prime targets for designing vaccines against this novel CoVs [118]. In addition, other conserved regions of SARS-CoV-2 including two subunits of the S-protein, M-protein as well as N-protein, can be applied as another potential target for design and development of effective vaccines.

Antiviral vaccines can be categorized into two broad groups: DNA- and RNA-based vaccines, in which individuals are injected with genetically engineered plasmid containing the DNA molecule encoding the antigen against which an immune response is eligible, thus the cells machinery creates the antigen, leading to immunological response; and peptide- or protein-based vaccines that include whole-inactivated virus, individual viral proteins or subdomains, and purified or recombinant proteinaceous antigens proteins from the virus, all of which are manufactured *in vitro*.

The candidate vaccines that have recently entered clinical development include: mRNA-1273, Ad5-nCoV, INO-4800 and LV-SMENP-DC and pathogen-specific aAPC (Table 2). Several platforms have progressed to development with potential for rapid development, including DNA- and RNA-based platforms, followed by those for developing recombinant-subunit vaccines. RNA and DNA vaccines can be made quickly because they do not require culture or fermentation, instead using synthetic processes [119,120].

**Table 2.** Vaccine in clinical trials for the treatment of coronavirus disease 2019 (updated 5 July 2020).

Intervention	Phase	Status	References
Pathogen-specific aAPC	I	Recruiting	NCT04299724
LV-SMENP-DC vaccine and antigen-specific CTLs	I/II	Recruiting	NCT04276896
– GX-19	I/II	Recruiting	NCT04445389
– Vaccine BCG	III	Not yet recruiting	NCT04362124
– Vaccine (mRNA-1273)	I	Recruiting	NCT04283461
– Novel coronavirus vaccine (adenovirus Type 5 vector)	I	Active, not recruiting	NCT04313127
– SCB-2019	I	Recruiting	NCT04405908
– Gam-COVID-Vac	I/II	Recruiting	NCT04436471
– Gam-COVID-Vac Lyo	I/II	Recruiting	NCT04437875
– Vaccine (adenovirus type 5 vector)	II	Active, not recruiting	NCT04341389
– BNT162a1	I	Recruiting	NCT04368728
– SARS-CoV-2 rS	I	Recruiting	NCT04368988
– AV-COVID-19	I/II	Not yet recruiting	NCT04386252
– Covax-19™	I	Not yet recruiting	NCT04428073
– Vaccine ChAdOx1 nCoV-19	II/III	Not yet recruiting	NCT04400838
– bacTRL-spike vaccine	I	Not yet recruiting	NCT04334980
– SARS-CoV-2 rS	I	Recruiting	NCT04368988
– IMM-101	III	Not yet recruiting	NCT04442048
– CVnCoV vaccine	I	Recruiting	NCT04449276
– ChAdOx1 nCoV-19	I/II	Not yet recruiting	NCT04444674
– VPM1002	III	Not yet recruiting	NCT04439045
– INO-4800	I/II	Not yet recruiting	NCT04447781
– INO-4800	I	Recruiting	NCT04336410
– BNT162a1	I/II	Recruiting	NCT04380701
– VPM1002	III	Recruiting	NCT04387409

aAPC: Artificial antigen-presenting cell; BCG: Bacille Calmette-Guérin or tuberculosis vaccine; CTL: Cytotoxic T lymphocyte.

Even with such promising platforms, SARS-CoV-2 vaccine development faces serious challenges. Although the virus’s S glycoprotein is a promising immunogen for protection, optimization of antigen design is crucial to obtaining an optimum host immune system response. Another concern is the possible exacerbation of lung disease, either directly or because of antibody-dependent enhancement due to the type 2 helper T-cell response. Furthermore, as with naturally acquired infection, the optimal duration of immunity is unknown; similarly, whether single-dose vaccines will confer lengthy immunity is doubtful.

## Conclusion

In the early phases of the epidemic, early detection assists management of the disease and preventive approaches such as masks, hand hygiene compliances, prevention of public contact, voluntary home quarantine, early diagnosis, contact tracing, intelligence social distance and travel restrictions have been recommended to decrease transmission. Other approaches include limiting events that may facilitate superspreader potential including religious services (marriages and funerals) [121]. Many dimensions of the SARS-CoV-2 and corresponding disease are unknown. For instance, the role of ACE2 receptors in SARS-CoV-2 pathogenesis remains uncertain. Future studies should be concentrate on profound understating of replication, pathogenesis and biological properties applying the relevant biological methods in other words reverse genetics and molecular techniques. Genome wide association studies may provide an opportunity for the identification of potential genetic factors contributed in the development of COVID-19. Although host genetic studies are expensive and complex, more studies are required to determine the role of host genetics (such as variation in HLA genes) in the immune response to CoVs, and the clinical outcome of CoVs-mediated disease. Understanding of the SAR-CoV-2 viral genetics during the time and geography specially regarding to the number and repetition of viral mutations and recombination rates and their association with viral infectivity, transmissibility, severity of disease and clinical manifestation, viral load and disease outcome are important knowledge gaps that navigate our research timetable. Until now, no unique antiviral therapy has been approved; so treatment is mainly based on symptomatic therapy and best supportive care.

The zoonotic link of SARS-CoV-2 infection has not been definitively proven; although, phylogenetic analysis shows that SARS-CoV-2 is very similar to SARS-like bat CoVs. Lessons from other human outbreaks from pathogenic viruses such as SARS-CoV, MERS-CoV and influenza viruses are very informative and valuable. Different wide-spectra antivirals agents previously used for treatment of influenza, SARS- and MERS-CoVs are under assessment for repurposing either monotherapy or in combinations to treat COVID-19 cases. SARS-CoV-2 is a novel human pathogen, and may interact with host antiviral defense via a specific pathway. Altogether, the infection and development of SARS-CoV-2 relies on the interplay between the virus and the patient’s immune response. Investigations of the area of SARS-CoV-2-host interplay provide response to many crucial questions in virus pathogenesis, disease control and prevention. At present, COVID-19 is leading to substantial global concerns. Development of valid, accurate and appropriate serological tests is urgently needed. It will be essential to quickly design and develop effective therapeutic regimen and vaccines to prevent or stop infection of this novel CoVs.

## Future perspective

The COVID-19 has caused more infections and deaths compared with either SARS or MERS. According to *R0* values, it is deemed that SARS-CoV-2 is more infectious than SARS or MERS. As imposition of globalization, CoVs will cause spreads and outbreaks with various mutant strains similarly in the coming years. With promotion of scientific collaboration, which is as a consequence of globalization, we may have more powerful means of combating CoVs, in which we characterize the genome structure and pathogenesis of SARS-CoV-2 infection very well in the near future. A present treatment is mainly supportive, but trials of vaccines and antivirals are in progress. Differences in the length of the spike as it is longer in SARS-CoV-2 are likely to play a major role in the pathogenesis and treatment of this virus.

Robust coordination and collaboration between researchers, vaccine developers, international regulators, policy-makers, financiers, national public health institutes and governments will be required to ensure that potential late-stage vaccine candidates can be produced in adequate amount with high safety and efficacy as well as equitably provided to all affected areas, specially low-resource regions.

**Executive summary**

**The severe acute respiratory syndrome coronavirus 2 genome & structure**

- All the coronaviruses are positive-stranded RNA viruses with a polycistronic genome with 6–11 open reading frames, encoding several nonstructural proteins at the 5′-end plus four structural proteins (spike surface glycoprotein [S], envelope [E], matrix [M] and nucleocapsid [N]) and multiple lineage-specific accessory proteins at the 3′-end.
- Phylogenetic analysis demonstrates that severe acute respiratory syndrome coronavirus 2 (SARS-CoV-2) shares 50 and 80.0% nucleotide identity to Middle East respiratory syndrome CoV and SARS-CoV, respectively.
- The S-protein of SARS-CoV-2 is longer than for other viruses such as SARS-CoV and Bat SARS-like CoVs.

**SARS-CoV-2 pathogenesis**

- Virion particles spread from the respiratory mucosa, by binding to the ACE2 receptors on ciliated bronchial epithelial cells, and after that may engage with other cells.
- The S-glycoprotein mediates binding of the virus to the sensitive human cell surface receptors, followed by fusion of the virus and host cell membranes to assist viral entrance.
- SARS-CoV-2 infection stimulates the immune response via two stages. At the incubation and nonalarming stages, a particular adaptive immune response is needed to eradicate the virus and to impede progress to severe condition.
- The injured cells activate innate inflammation within the lungs, which is mainly mediated through pro-inflammatory macrophages/monocytes. Lung inflammation is the major reason for the fatal respiratory disease at the severe stage of coronavirus disease 2019 (COVID-19) infection.
- The main characteristics of COVID-19 on preliminary computed tomography (CT) examination including bilateral multi-lobar ground-glass opacities with a peripheral/posterior distribution and patchy consolidation.

**Diagnosis & treatment of COVID-19**

- At present, the diagnosis of COVID-19 is largely based on laboratory tests PCR and chest CT imaging technique. Although, no specific antiviral treatment for COVID-19 is currently advised due to lack of evidence.
- Several pharmacotherapeutic agents have been used for treatment of COVID-19 patients consisting lopinavir/ritonavir, hydroxychloroquine and IFN β-1A.
- Effective SARS-CoV-2 vaccines are urgently needed in order to decrease infection severity, viral shedding as well as human–human transmission. The most advanced candidates have recently moved into clinical development, including mRNA-1273, Ad5-nCoV, INO-4800 and LV-SMENP-DC, and pathogen-specific.

## References

[1] de Wit E, van Doremalen N, Falzarano D, Munster VJ SARS and MERS: recent insights into emerging coronaviruses. Nat. Rev. Microbiol. 14(8), 523 (2016). 27344959 10.1038/nrmicro.2016.81 PMC7097822

[2] Menachery VD, Graham RL, Baric RS Jumping species – a mechanism for coronavirus persistence and survival. Curr. Opin. Virol. 23, 1–7 (2017). 28214731 10.1016/j.coviro.2017.01.002 PMC5474123

[3] Fung S-Y, Yuen K-S, Ye Z-W, Chan C-P, Jin D-Y A tug-of-war between severe acute respiratory syndrome coronavirus 2 and host antiviral defence: lessons from other pathogenic viruses. Emerg. Microbes Infect. 9(1), 558–570 (2020). 32172672 10.1080/22221751.2020.1736644 PMC7103735

[4] Xu X, Chen P, Wang J Evolution of the novel coronavirus from the ongoing Wuhan outbreak and modeling of its spike protein for risk of human transmission. Sci. China Life Sci. 63(3), 457–460 (2020). 32009228 10.1007/s11427-020-1637-5 PMC7089049

[5] Wong JE, Leo YS, Tan CC COVID-19 in Singapore – current experience: critical global issues that require attention and action. JAMA (2020) (Epub ahead of print). 10.1001/jama.2020.2467 32077901

[6] Thompson R Pandemic potential of 2019-nCoV. Lancet Infect. Dis. 20(3), 280 (2020). 10.1016/S1473-3099(20)30068-2 PMC7128127 32043978

[7] Peiris J, Lai S, Poon L Coronavirus as a possible cause of severe acute respiratory syndrome. Lancet 361(9366), 1319–1325 (2003). 12711465 10.1016/S0140-6736(03)13077-2 PMC7112372

[8] Zhu N, Zhang D, Wang W A novel coronavirus from patients with pneumonia in China, 2019. N. Engl. J. Med. 382(8), 727–733 (2020). 31978945 10.1056/NEJMoa2001017 PMC7092803

[9] Zhou P, Fan H, Lan T Fatal swine acute diarrhoea syndrome caused by an HKU2-related coronavirus of bat origin. Nature 556(7700), 255–258 (2018). 29618817 10.1038/s41586-018-0010-9 PMC7094983

[10] Zou L, Ruan F, Huang M SARS-CoV-2 viral load in upper respiratory specimens of infected patients. N. Engl. J. Med. 382(12), 1177–1179 (2020). 32074444 10.1056/NEJMc2001737 PMC7121626

[11] Liu Y, Gayle AA, Wilder-Smith A, Rocklöv J The reproductive number of COVID-19 is higher compared to SARS coronavirus. J. Travel Med. 27(2), (2020). 10.1093/jtm/taaa021 PMC7074654 32052846

[12] Deng X, van Geelen A, Buckley AC Coronavirus endoribonuclease activity in porcine epidemic diarrhea virus suppresses type I and type III interferon responses. J. Virol. 93(8), e02000–02018 (2019). 30728254 10.1128/JVI.02000-18 PMC6450110

[13] Zhou P, Yang X-L, Wang X-G A pneumonia outbreak associated with a new coronavirus of probable bat origin. Nature 579(7798), 270–273 (2020). 32015507 10.1038/s41586-020-2012-7 PMC7095418

[14] Wu F, Zhao S, Yu B A new coronavirus associated with human respiratory disease in China. Nature 579(7798), 265–269 (2020). 32015508 10.1038/s41586-020-2008-3 PMC7094943

[15] Siu Y, Teoh K, Lo J The M, E, and N structural proteins of the severe acute respiratory syndrome coronavirus are required for efficient assembly, trafficking, and release of virus-like particles.. J. Virol. 82(22), 11318–11330 (2008). 18753196 10.1128/JVI.01052-08 PMC2573274

[16] Kirchdoerfer RN, Cottrell CA, Wang N Pre-fusion structure of a human coronavirus spike protein. Nature 531(7592), 118–121 (2016). 26935699 10.1038/nature17200 PMC4860016

[17] Lu G, Wang Q, Gao GF Bat-to-human: spike features determining ‘host jump’ of coronaviruses SARS-CoV, MERS-CoV, and beyond. Trends Microbiol. 23(8), 468–478 (2015). 26206723 10.1016/j.tim.2015.06.003 PMC7125587

[18] Millet JK, Whittaker GR Host cell entry of Middle East respiratory syndrome coronavirus after two-step, furin-mediated activation of the spike protein. Proc. Natl Acad. Sci. USA 111(42), 15214–15219 (2014). 25288733 10.1073/pnas.1407087111 PMC4210292

[19] Li F Structure, function, and evolution of coronavirus spike proteins. Annu. Rev. Virol. 3(1), 237–261 (2016). 27578435 10.1146/annurev-virology-110615-042301 PMC5457962

[20] Zhu Z, Zhang Z, Chen W Predicting the receptor-binding domain usage of the coronavirus based on kmer frequency on spike protein. Infect. Genet. Evol. 61, 183 (2018). 29625240 10.1016/j.meegid.2018.03.028 PMC7129160

[21] Belouzard S, Millet JK, Licitra BN, Whittaker GR Mechanisms of coronavirus cell entry mediated by the viral spike protein. Viruses 4(6), 1011–1033 (2012). 22816037 10.3390/v4061011 PMC3397359

[22] Wrapp D, Wang N, Corbett KS Cryo-EM structure of the 2019-nCoV spike in the prefusion conformation. Science 367(6483), 1260–1263 (2020). 32075877 10.1126/science.abb2507 PMC7164637

[23] Fehr AR, Perlman S Coronaviruses: an overview of their replication and pathogenesis. : Coronaviruses Methods in Molecular Biology. Maier H, Bickerton E, Britton P (Eds). Springer, NY, USA, 1282, 1–23 (2015). 10.1007/978-1-4939-2438-7_1 PMC4369385 25720466

[24] Glowacka I, Bertram S, Müller MA Evidence that TMPRSS2 activates the severe acute respiratory syndrome coronavirus spike protein for membrane fusion and reduces viral control by the humoral immune response. J. Virol. 85(9), 4122–4134 (2011). 21325420 10.1128/JVI.02232-10 PMC3126222

[25] Qian Z, Dominguez SR, Holmes KV Role of the spike glycoprotein of human Middle East respiratory syndrome coronavirus (MERS-CoV) in virus entry and syncytia formation. PLoS ONE 8(10), e76469 (2013). 24098509 10.1371/journal.pone.0076469 PMC3789674

[26] Li W, Moore MJ, Vasilieva N Angiotensin-converting enzyme 2 is a functional receptor for the SARS coronavirus. Nature 426(6965), 450–454 (2003). 14647384 10.1038/nature02145 PMC7095016

[27] Matsuyama S, Nagata N, Shirato K, Kawase M, Takeda M, Taguchi F Efficient activation of the severe acute respiratory syndrome coronavirus spike protein by the transmembrane protease TMPRSS2. J. Virol. 84(24), 12658–12664 (2010). 20926566 10.1128/JVI.01542-10 PMC3004351

[28] Wan Y, Shang J, Graham R, Baric RS, Li F Receptor recognition by the novel coronavirus from Wuhan: an analysis based on decade-long structural studies of SARS coronavirus. J. Virol. 94(7), e00127–20 (2020). 31996437 10.1128/JVI.00127-20 PMC7081895

[29] Gralinski LE, Menachery VD Return of the coronavirus: 2019-nCoV. Viruses 12(2), 135 (2020). 10.3390/v12020135 PMC7077245 31991541

[30] Mortola E, Roy P Efficient assembly and release of SARS coronavirus-like particles by a heterologous expression system. FEBS Lett. 576(1–2), 174–178 (2004). 15474033 10.1016/j.febslet.2004.09.009 PMC7126153

[31] Corse E, Machamer CE Infectious bronchitis virus E protein is targeted to the Golgi complex and directs release of virus-like particles. J. Virol. 74(9), 4319–4326 (2000). 10756047 10.1128/jvi.74.9.4319-4326.2000 PMC111949

[32] Corse E, Machamer CE The cytoplasmic tails of infectious bronchitis virus E and M proteins mediate their interaction. Virology 312(1), 25–34 (2003). 12890618 10.1016/S0042-6822(03)00175-2 PMC7127533

[33] Neuman BW, Kiss G, Kunding AH A structural analysis of M protein in coronavirus assembly and morphology. J. Struct. Biol. 174(1), 11–22 (2011). 21130884 10.1016/j.jsb.2010.11.021 PMC4486061

[34] de Haan CA, Rottier PJ Molecular interactions in the assembly of coronaviruses. Adv. Virus Res. 64, 165–230 (2005). 16139595 10.1016/S0065-3527(05)64006-7 PMC7112327

[35] Escors D, Ortego J, Laude H, Enjuanes L The membrane M protein carboxy terminus binds to transmissible gastroenteritis coronavirus core and contributes to core stability. J. Virol. 75(3), 1312–1324 (2001). 11152504 10.1128/JVI.75.3.1312-1324.2001 PMC114037

[36] Narayanan K, Maeda A, Maeda J, Makino S Characterization of the coronavirus M protein and nucleocapsid interaction in infected cells. J. Virol. 74(17), 8127–8134 (2000). 10933723 10.1128/jvi.74.17.8127-8134.2000 PMC112346

[37] Cui J, Li F, Shi Z-L Origin and evolution of pathogenic coronaviruses. Nat. Rev. Microbiol. 17(3), 181–192 (2019). 30531947 10.1038/s41579-018-0118-9 PMC7097006

[38] Lu R, Zhao X, Li J Genomic characterisation and epidemiology of 2019 novel coronavirus: implications for virus origins and receptor binding. Lancet 395(10224), 565–574 (2020). 32007145 10.1016/S0140-6736(20)30251-8 PMC7159086

[39] Calisher C, Carroll D, Colwell R Statement in support of the scientists, public health professionals, and medical professionals of China combatting COVID-19. Lancet 395(10226), e42–e43 (2020). 32087122 10.1016/S0140-6736(20)30418-9 PMC7159294

[40] Zhu N, Zhang D, Wang W China Novel Coronavirus Investigating and Research Team. A novel coronavirus from patients with pneumonia in China, 2019. N. Engl. J. Med. 382(8), 727–733 (2020). 31978945 10.1056/NEJMoa2001017 PMC7092803

[41] Chan JF-W, Kok K-H, Zhu Z Genomic characterization of the 2019 novel human-pathogenic coronavirus isolated from a patient with atypical pneumonia after visiting Wuhan. Emerg. Microbes Infect. 9(1), 221–236 (2020). 31987001 10.1080/22221751.2020.1719902 PMC7067204

[42] Zhou P, Yang X-L, Wang X-G Discovery of a novel coronavirus associated with the recent pneumonia outbreak in humans and its potential bat origin. BioRxiv. 2020) (Epub ahead of print).

[43] Paraskevis D, Kostaki EG, Magiorkinis G, Panayiotakopoulos G, Sourvinos G, Tsiodras S Full-genome evolutionary analysis of the novel corona virus (2019-nCoV) rejects the hypothesis of emergence as a result of a recent recombination event. Infect. Genet. Evol. 79, 104212 (2020). 32004758 10.1016/j.meegid.2020.104212 PMC7106301

[44] Coutard B, Valle C, de Lamballerie X, Canard B, Seidah N, Decroly E The spike glycoprotein of the new coronavirus 2019-nCoV contains a furin-like cleavage site absent in CoV of the same clade. Antiviral Res. 176, 104742 (2020). 32057769 10.1016/j.antiviral.2020.104742 PMC7114094

[45] Wu F, Zhao S, Yu B Complete genome characterisation of a novel coronavirus associated with severe human respiratory disease in Wuhan, China. BioRxiv 2020) (Epub ahead of print).

[46] Angeletti S, Benvenuto D, Bianchi M, Giovanetti M, Pascarella S, Ciccozzi M COVID-2019: the role of the nsp2 and nsp3 in its pathogenesis.. J. Med. Virol. 92(6), 584–588 (2020). 32083328 10.1002/jmv.25719 PMC7228367

[47] Saikatendu KS, Joseph JS, Subramanian V Structural basis of severe acute respiratory syndrome coronavirus ADP-ribose-1″-phosphate dephosphorylation by a conserved domain of nsP3. Structure 13(11), 1665–1675 (2005). 16271890 10.1016/j.str.2005.07.022 PMC7126892

[48] Shang J, Ye G, Shi K Structural basis for receptor recognition by the novel coronavirus from Wuhan. Research Square 2020) (Epub ahead of print).

[49] Letko MC, Munster V Functional assessment of cell entry and receptor usage for lineage B β-coronaviruses, including 2019-nCoV. BioRxiv 2020) (Epub ahead of print). 10.1038/s41564-020-0688-y PMC7095430 32094589

[50] D'Amico F, Baumgart DC, Danese S, Peyrin-Biroulet L Diarrhea during COVID-19 infection: pathogenesis, epidemiology, prevention and management. Clin. Gastroenterol. Hepatol. 18(8), 1663–1672 (2020). 32278065 10.1016/j.cgh.2020.04.001 PMC7141637

[51] Isabel S, Grana-Miraglia L, Gutierrez JM Evolutionary and structural analyses of SARS-CoV-2 D614G spike protein mutation now documented worldwide. BioRxiv 2020) (Epub ahead of print). 10.1038/s41598-020-70827-z PMC7441380 32820179

[52] Hu J, He CL, Gao Q The D614G mutation of SARS-CoV-2 spike protein enhances viral infectivity. BioRxiv 2020) (Epub ahead of print).

[53] Bhattacharyya C, Das C, Ghosh A Global spread of SARS-CoV-2 subtype with spike protein mutation D614G is shaped by human genomic variations that regulate expression of TMPRSS2 and MX1 genes. BioRxiv 2020) (Epub ahead of print).

[54] Daniloski Z, Guo X, Sanjana NE The D614G mutation in SARS-CoV-2 Spike increases transduction of multiple human cell types. BioRxiv 2020) (Epub ahead of print). 10.7554/eLife.65365 PMC7891930 33570490

[55] Knoops K, Kikkert M, van den Worm SH SARS-coronavirus replication is supported by a reticulovesicular network of modified endoplasmic reticulum. PLoS Biol. 6(9), 1957–1974 (2008). 10.1371/journal.pbio.0060226 PMC2535663 18798692

[56] Müller C, Hardt M, Schwudke D, Neuman BW, Pleschka S, Ziebuhr J Inhibition of cytosolic phospholipase A2α impairs an early step of coronavirus replication in cell culture. J. Virol. 92(4), e01463–01417 (2018). 29167338 10.1128/JVI.01463-17 PMC5790932

[57] Vijay R, Hua X, Meyerholz DK Critical role of phospholipase A2 group IID in age-related susceptibility to severe acute respiratory syndrome–CoV infection. J. Exp. Med. 212(11), 1851–1868 (2015). 26392224 10.1084/jem.20150632 PMC4612096

[58] Jaimes JA, Andre NM, Millet JK, Whittaker GR Structural modeling of 2019-novel coronavirus (nCoV) spike protein reveals a proteolytically-sensitive activation loop as a distinguishing feature compared to SARS-CoV and related SARS-like coronaviruses. Preprint. 1–36 (2020).

[59] Ou X, Liu Y, Lei X Characterization of spike glycoprotein of SARS-CoV-2 on virus entry and its immune cross-reactivity with SARS-CoV. Nat. Commun. 11(1), 1–12 (2020). 32221306 10.1038/s41467-020-15562-9 PMC7100515

[60] Qin C, Zhou L, Hu Z Dysregulation of immune response in patients with COVID-19 in Wuhan, China. Clin. Infect. Dis. (2020) (Epub ahead of print). 10.1093/cid/ciaa248 PMC7108125 32161940

[61] Xu Z, Shi L, Wang Y Pathological findings of COVID-19 associated with acute respiratory distress syndrome. Lancet Respir. Med. 8(4), 420–422 (2020). 32085846 10.1016/S2213-2600(20)30076-X PMC7164771

[62] Tian S, Hu W, Niu L, Liu H, Xu H, Xiao S-Y Pulmonary pathology of early phase 2019 novel coronavirus (COVID-19) pneumonia in two patients with lung cancer. J. Thorac. Oncol. 15(5), 700–704 (2020). 32114094 10.1016/j.jtho.2020.02.010 PMC7128866

[63] Zhou Y, Fu B, Zheng X, Wang D, Zhao C Pathogenic T cells and inflammatory monocytes incite inflammatory storm in severe COVID-19 patients. Natl Sci. Rev. 7(6), 998–1002 (2020). 10.1093/nsr/nwaa041 PMC7108005 34676125

[64] Moossavi M, Parsamanesh N, Bahrami A, Atkin SL, Sahebkar A Role of the NLRP3 inflammasome in cancer. Mol. Cancer 17(1), 158 (2018). 30447690 10.1186/s12943-018-0900-3 PMC6240225

[65] Parsamanesh N, Moossavi M, Bahrami A, Fereidouni M, Barreto G, Sahebkar A NLRP3 inflammasome as a treatment target in atherosclerosis: a focus on statin therapy. Int. Immunopharmacol. 73, 146–155 (2019). 31100709 10.1016/j.intimp.2019.05.006

[66] Shi C-S, Nabar NR, Huang N-N, Kehrl JH SARS-coronavirus open reading frame-8b triggers intracellular stress pathways and activates NLRP3 inflammasomes. Cell Death Discov. 5(1), 1–12 (2019). 10.1038/s41420-019-0181-7 PMC6549181 31231549

[67] Shi Y, Wang Y, Shao C COVID-19 infection: the perspectives on immune responses. Cell Death Differ. 27(5), 1451–1454 (2020). 32205856 10.1038/s41418-020-0530-3 PMC7091918

[68] Xu Z, Shi L, Wang Y Pathological findings of COVID-19 associated with acute respiratory distress syndrome. Lancet Respir. Med. 8(4), 420–422 (2020). 32085846 10.1016/S2213-2600(20)30076-X PMC7164771

[69] Sardar R, Satish D, Birla S, Gupta D Comparative analyses of SAR-CoV2 genomes from different geographical locations and other coronavirus family genomes reveals unique features potentially consequential to host-virus interaction and pathogenesis. BioRxiv 2020) (Epub ahead of print). 10.1016/j.heliyon.2020.e04658 PMC7439967 32844125

[70] Rodriguez-Morales AJ, Cardona-Ospina JA, Gutiérrez-Ocampo E Clinical, laboratory and imaging features of COVID-19: a systematic review and meta-analysis. Travel Med. Infect. Dis. 34, 101623 (2020). 32179124 10.1016/j.tmaid.2020.101623 PMC7102608

[71] Li Q, Guan X, Wu P Early transmission dynamics in Wuhan, China, of novel coronavirus–infected pneumonia. N. Engl. J. Med. 382(13), 1199–1207 (2020). 31995857 10.1056/NEJMoa2001316 PMC7121484

[72] Linton NM, Kobayashi T, Yang Y Incubation period and other epidemiological characteristics of 2019 novel coronavirus infections with right truncation: a statistical analysis of publicly available case data. J. Clin. Med. 9(2), 538 (2020). 10.3390/jcm9020538 PMC7074197 32079150

[73] Chen N, Zhou M, Dong X Epidemiological and clinical characteristics of 99 cases of 2019 novel coronavirus pneumonia in Wuhan, China: a descriptive study. Lancet 395(10223), 507–513 (2020). 32007143 10.1016/S0140-6736(20)30211-7 PMC7135076

[74] Huang C, Wang Y, Li X Clinical features of patients infected with 2019 novel coronavirus in Wuhan, China. Lancet 395(10223), 497–506 (2020). 31986264 10.1016/S0140-6736(20)30183-5 PMC7159299

[75] Guan W-J, Ni Z-y, Hu Y Clinical characteristics of coronavirus disease 2019 in China. N. Engl. J. Med. 382, 1708–1720 (2020). 32109013 10.1056/NEJMoa2002032 PMC7092819

[76] Salehi S, Abedi A, Balakrishnan S, Gholamrezanezhad A Coronavirus disease 2019 (COVID-19): a systematic review of imaging findings in 919 patients. AJR Am. J. Roentgenol. 215, 87–93 (2020). 32174129 10.2214/AJR.20.23034

[77] Lippi G, Plebani M Laboratory abnormalities in patients with COVID-2019 infection. Clin. Chem. Lab. Med. 58(7), 1131–1134 (2020). 32119647 10.1515/cclm-2020-0198

[78] Chau TN, Lee KC, Yao H SARS-associated viral hepatitis caused by a novel coronavirus: report of three cases. Hepatology 39(2), 302–310 (2004). 14767982 10.1002/hep.20111 PMC7165792

[79] Shi S, Qin M, Shen B Association of cardiac injury with mortality in hospitalized patients with COVID-19 in Wuhan, China. JAMA Cardiol. (2020) (Epub ahead of print). 10.1001/jamacardio.2020.0950 PMC7097841 32211816

[80] Guan W-J, Ni Z-y, Hu Y Clinical characteristics of coronavirus disease 2019 in China. N. Engl. J. Med. 382(18), 1708–1720 (2020). 32109013 10.1056/NEJMoa2002032 PMC7092819

[81] Zhang J, Dong X, Cao Y-Y Clinical characteristics of 140 patients infected with SARS-CoV-2 in Wuhan, China. Allergy 75(7), 1730–1741 (2020). 32077115 10.1111/all.14238

[82] Shi Y, Yu X, Zhao H, Wang H, Zhao R, Sheng J Host susceptibility to severe COVID-19 and establishment of a host risk score: findings of 487 cases outside Wuhan. Crit. Care 24(1), 1–4 (2020). 32188484 10.1186/s13054-020-2833-7 PMC7081524

[83] Liang W, Guan W, Chen R Cancer patients in SARS-CoV-2 infection: a nationwide analysis in China. Lancet Oncol. 21(3), 335–337 (2020). 32066541 10.1016/S1470-2045(20)30096-6 PMC7159000

[84] He X, Lau EH, Wu P Temporal dynamics in viral shedding and transmissibility of COVID-19. Nat. Med. 26(5), 672–675 (2020). 32296168 10.1038/s41591-020-0869-5

[85] Liu Y, Yan L-M, Wan L Viral dynamics in mild and severe cases of COVID-19. Lancet Infect. Dis. 20(6), 656–657 (2020). 32199493 10.1016/S1473-3099(20)30232-2 PMC7158902

[86] Shi H, Han X, Jiang N Radiological findings from 81 patients with COVID-19 pneumonia in Wuhan, China: a descriptive study. Lancet Infect. Dis. 20(4), 425–434 (2020). 32105637 10.1016/S1473-3099(20)30086-4 PMC7159053

[87] WHO. Laboratory testing for coronavirus disease 2019 (COVID-19) in suspected human cases: interim guidance (2020). https://apps.who.int/iris/handle/10665/331329

[88] WHO. Coronavirus disease 2019 (COVID-19): situation report – 28. (2020). www.who.int/docs/default-source/coronaviruse/situation-reports/20200217-sitrep-28-covid-19.pdf?sfvrsn=a19cf2ad_2

[89] Chung M, Bernheim A, Mei X CT imaging features of 2019 novel coronavirus (2019-nCoV). Radiology 295(1), 202–207 (2020). 32017661 10.1148/radiol.2020200230 PMC7194022

[90] Pan Y, Zhang D, Yang P, Poon LL, Wang Q Viral load of SARS-CoV-2 in clinical samples. Lancet Infect. Dis. 20(4), 411–412 (2020). 32105638 10.1016/S1473-3099(20)30113-4 PMC7128099

[91] Wölfel R, Corman VM, Guggemos W Virological assessment of hospitalized patients with COVID-2019. Nature 581(7809), 465–469 (2020). 32235945 10.1038/s41586-020-2196-x

[92] Li T, Wei C, Li W, Hongwei F, Shi J Beijing Union Medical College Hospital on ‘pneumonia of novel coronavirus infection’ diagnosis and treatment proposal (V2. 0). Med. J. Peking Union Med. Coll. Hosp. (2020). http://kns.cnki.net/kcms/detail/11.5882.r.20200130.1430.002.html

[93] Kim C, Ahmed JA, Eidex RB Comparison of nasopharyngeal and oropharyngeal swabs for the diagnosis of eight respiratory viruses by real-time reverse transcription-PCR assays. PLoS ONE 6(6), e21610 (2011). 21738731 10.1371/journal.pone.0021610 PMC3128075

[94] Guo L, Ren L, Yang S Profiling early humoral response to diagnose novel coronavirus disease (COVID-19). Clin. Infect. Dis. 71(15), 778–785 (2020). 32198501 10.1093/cid/ciaa310 PMC7184472

[95] Rashid ZZ, Othman SN, Samat MNA, Ali UK, Wong KK Diagnostic performance of COVID-19 serology assays. Malays. J. Pathol. 42(1), 13–21 (2020). 32342927

[96] WHO. Clinical management of severe acute respiratory infection when novel coronavirus (nCoV) infection is suspected: Interim guidance, 13 March 2020 (2020). https://apps.who.int/iris/handle/10665/331446

[97] WHO. Clinical management of severe acute respiratory infection when novel coronavirus (nCoV) infection is suspected: interim guidance, 25 January 2020 (2020). https://apps.who.int/iris/bitstream/handle/10665/330854/WHO-nCoV-Clinical-2020.2-eng.pdf?sequence=1&isAllowed=y

[98] Wang M, Cao R, Zhang L Remdesivir and chloroquine effectively inhibit the recently emerged novel coronavirus (2019-nCoV) *in vitro*. Cell Res. 30(3), 269–271 (2020). 32020029 10.1038/s41422-020-0282-0 PMC7054408

[99] Gautret P, Lagier J-C, Parola P Hydroxychloroquine and azithromycin as a treatment of COVID-19: results of an open-label non-randomized clinical trial. Int. J. Antimicrob. Agents. (2020) (Epub ahead of print). 10.1016/j.ijantimicag.2020.105949 PMC7102549 32205204

[100] Cortegiani A, Ingoglia G, Ippolito M, Giarratano A, Einav S A systematic review on the efficacy and safety of chloroquine for the treatment of COVID-19. J. Crit. Care 57, 279–283 (2020). 32173110 10.1016/j.jcrc.2020.03.005 PMC7270792

[101] Chen J, LIU D, LIU L A pilot study of hydroxychloroquine in treatment of patients with common coronavirus disease-19 (COVID-19). J. Zhejiang University (Medical Science) 49(2), 215–219 (2020). 10.3785/j.issn.1008-9292.2020.03.03 PMC8800713 32391667

[102] Molina JM, Delaugerre C, Le Goff J No evidence of rapid antiviral clearance or clinical benefit with the combination of hydroxychloroquine and azithromycin in patients with severe COVID-19 infection. Med. Mal. Infect. 50(384), 30085–30088 (2020). 10.1016/j.medmal.2020.03.006 PMC7195369 32240719

[103] Cao B, Wang Y, Wen D A trial of lopinavir–ritonavir in adults hospitalized with severe Covid-19. N. Engl. J. Med. 382, 1787–1799 (2020). 32187464 10.1056/NEJMoa2001282 PMC7121492

[104] Zhu Z, Lu Z, Xu T Arbidol monotherapy is superior to lopinavir/ritonavir in treating COVID-19. J. Infect. 81(1), e21–e23 (2020). 32283143 10.1016/j.jinf.2020.03.060 PMC7195393

[105] Xu Z, Yao H, Shen J Nelfinavir is active against SARS-CoV-2 in Vero E6 cells. ChemRxiv 2020) (Epub ahead of print).

[106] Xu Z, Peng C, Shi Y Nelfinavir was predicted to be a potential inhibitor of 2019-nCov main protease by an integrative approach combining homology modelling, molecular docking and binding free energy calculation. BioRxiv 2020) (Epub ahead of print).

[107] Jin Z, Du X, Xu Y Structure of Mpro from COVID-19 virus and discovery of its inhibitors. BioRxiv 2020) (Epub ahead of print).

[108] Li S-y, Chen C, Zhang H-q Identification of natural compounds with antiviral activities against SARS-associated coronavirus. Antiviral Res. 67(1), 18–23 (2005). 15885816 10.1016/j.antiviral.2005.02.007 PMC7114104

[109] Lin C-W, Tsai F-J, Tsai C-H Anti-SARS coronavirus 3C-like protease effects of Isatis indigotica root and plant-derived phenolic compounds. Antiviral Res. 68(1), 36–42 (2005). 16115693 10.1016/j.antiviral.2005.07.002 PMC7114321

[110] Lau K-M, Lee K-M, Koon C-M Immunomodulatory and anti-SARS activities of Houttuynia cordata. J. Ethnopharmacol. 118(1), 79–85 (2008). 18479853 10.1016/j.jep.2008.03.018 PMC7126383

[111] Adem S, Eyupoglu V, Sarfraz I, Rasul A, Ali M Identification of potent COVID-19 main protease (Mpro) inhibitors from natural polyphenols: an *in silico* strategy unveils a hope against CORONA. Preprints 2020) (Epub ahead of print). 10.1016/j.compbiomed.2022.105452 PMC8957318 35364308

[112] Tian X, Li C, Huang A Potent binding of 2019 novel coronavirus spike protein by a SARS coronavirus-specific human monoclonal antibody. Emerg. Microbes. Infect. 9(1), 382–385 (2020). 32065055 10.1080/22221751.2020.1729069 PMC7048180

[113] Elfiky AA Anti-HCV, nucleotide inhibitors, repurposing against COVID-19. Life Sci. 248, 117477 (2020). 32119961 10.1016/j.lfs.2020.117477 PMC7089605

[114] Holshue ML, DeBolt C, Lindquist S First case of 2019 novel coronavirus in the United States. N. Engl. J. Med. 382, 929–936 (2020). 32004427 10.1056/NEJMoa2001191 PMC7092802

[115] Roback JD, Guarner J Convalescent plasma to treat COVID-19: possibilities and challenges. JAMA 323(16), 1561–1562 (2020). 10.1001/jama.2020.4940 32219429

[116] Mair-Jenkins J, Saavedra-Campos M, Baillie JK The effectiveness of convalescent plasma and hyperimmune immunoglobulin for the treatment of severe acute respiratory infections of viral etiology: a systematic review and exploratory meta-analysis. J. Infect. Dis. 211(1), 80–90 (2015). 25030060 10.1093/infdis/jiu396 PMC4264590

[117] Tanne JH Covid-19: FDA approves use of convalescent plasma to treat critically ill patients. BMJ 368, m1256 (2020). 32217555 10.1136/bmj.m1256

[118] Jiang S, Du L, Shi Z An emerging coronavirus causing pneumonia outbreak in Wuhan, China: calling for developing therapeutic and prophylactic strategies. Emerg. Microbes Infect. 9(1), 275–277 (2020). 32005086 10.1080/22221751.2020.1723441 PMC7033706

[119] Le TT, Andreadakis Z, Kumar A The COVID-19 vaccine development landscape. Nat. Rev. Drug Discov. 19(5), 305–306 (2020). 32273591 10.1038/d41573-020-00073-5

[120] Lurie N, Saville M, Hatchett R, Halton J Developing Covid-19 vaccines at pandemic speed. N. Engl. J. Med. 382(21), 1969–1973 (2020). 32227757 10.1056/NEJMp2005630

[121] Ebrahim SH, Ahmed QA, Gozzer E, Schlagenhauf P, Memish ZA Covid-19 and community mitigation strategies in a pandemic. BMJ 368, m1066 (2020). 32184233 10.1136/bmj.m1066

